# Acute intestinal obstruction revealing synchronous gastrointestinal stromal tumors in a small bowel diverticulum and mucinous adenocarcinoma of the colon: a case report

**DOI:** 10.11604/pamj.2015.21.172.2828

**Published:** 2015-07-02

**Authors:** El Ochi Mohamed Reda, Jahid Ahmed, El Ktaibi Abderrahim, Znati Kawtar, Zouaidia Fouad, Bernoussi Zakia, Mahassini Najat

**Affiliations:** 1 Department of Pathology, Ibn Sina University Hospital, Rabat, Morocco

**Keywords:** Intestinal obstruction, stromal tumors, colon

## Abstract

Gastrointestinalstromal tumors are rare neoplasms and represent 0,1% to 3% of all gastrointestinal cancers. They are the most frequent mesenchymal neoplasms of the gastrointestinal tract with a malignant potential and unpredictable behavior. The synchronous association with other primary gastrointestinal carcinoma has been rarely reported in the literature with increasing number in the last ten years. The associated Gastrointestinalstromal tumor is usually discovered incidentally during surgery for carcinoma. The limited number of these cases cannot confirm the existence of a common factor in tumorigenesis of these different tumors and other studies are needed to clarify the possible association. We report the first case in the literature of synchronous primary Gastrointestinalstromal tumors developed in small bowel diverticulum and mucinous adenocarcinoma of the colon. Key words: Synchronous, Gastrointestinalstromal tumors, Adenocarcinoma, Colon.

## Introduction

Gastrointestinalstromal tumors (GIST) are rare mesenchymal tumors of the gastrointestinal tract with an incidence of 1,5/100000 habitant/year [1]. They occur in adults especially in the sixth an seventh decade [2]. The concomitant association with other primary gastrointestinal malignancy has been rarely reported. Most of these publications describe gastric stromal tumors synchronous with another gastric malignancy [3]. We report a 60 year old male with synchronous mucinous adenocarcinoma of the colon and gastrointestinal stromal tumor in small bowel diverticulum.

## Patient and observation

**Figure 1:**
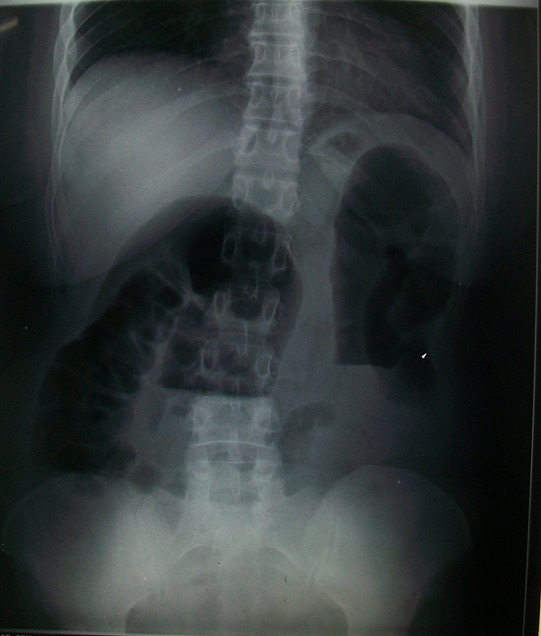
Abdominal X-ray showing the presence of air-fluid levels

**Figure 2:**
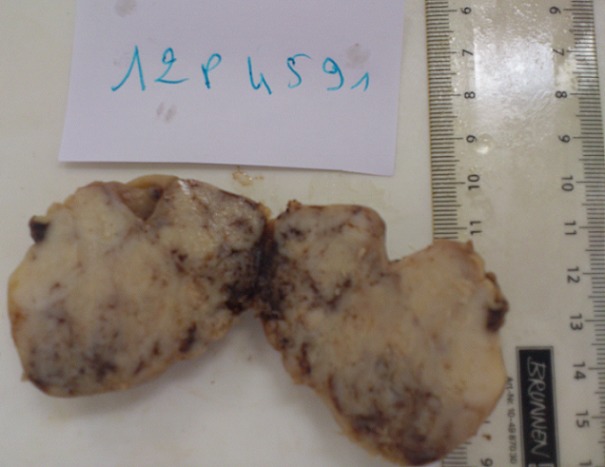
Macroscopic appearance of GIST in small bowel diverticulum

**Figure 3:**
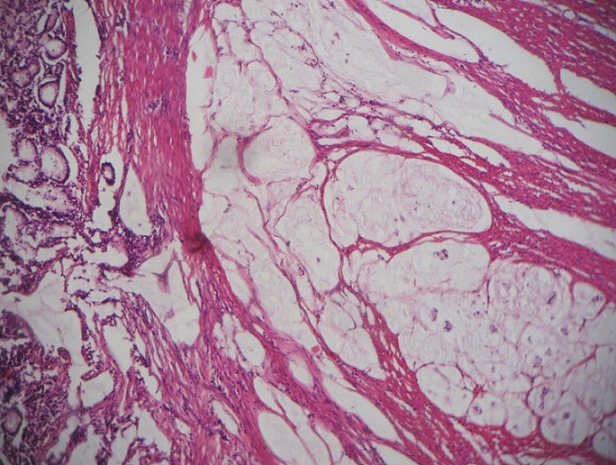
Mucinous adenocarcinoma of the sigmoid (HE stain, ob. x 20)

**Figure 4:**
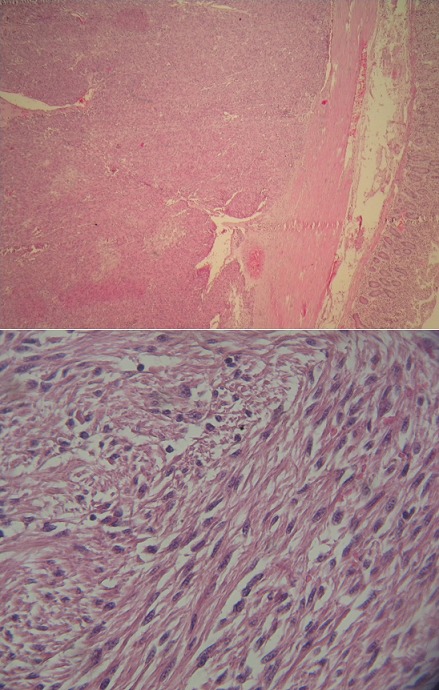
Fusiform low grade GIST: (A) invasion of submucosa of small intestine from GIST (HE stain, ob. x 10); (B) the GIST was composed of fascicles of spindle cell with no atypia (HE stain, ob. x 40)

**Figure 5:**
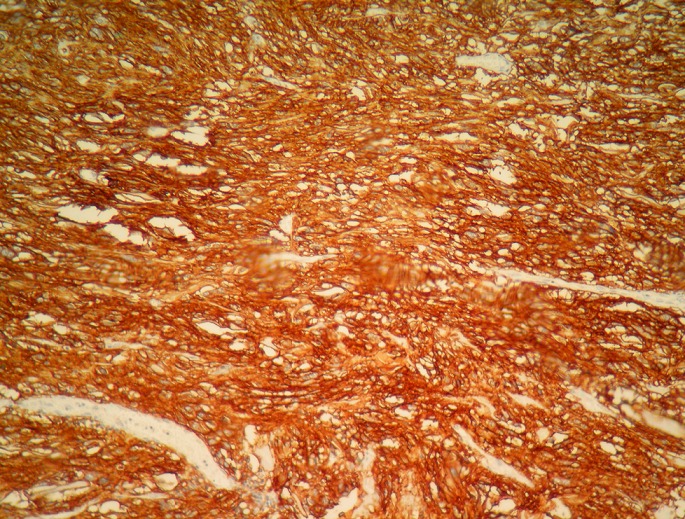
GIST: strong positive CD117 immunostaining (ob. x 40)

**Figure 6:**
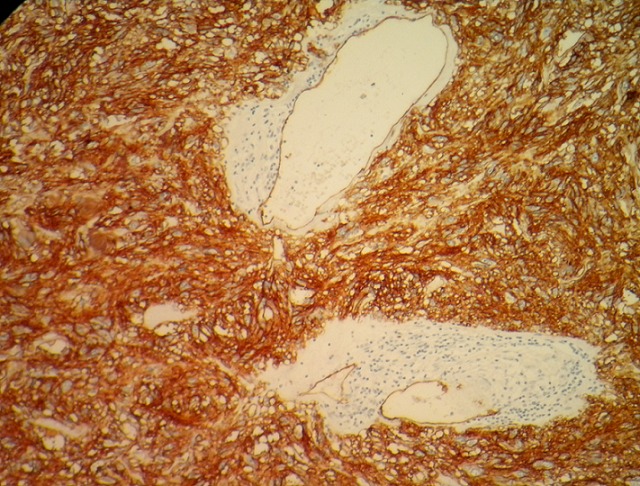
GIST: CD34 positivity (ob. x 40)

A 60 year old male without clinical antecedents was admitted to the emergency room complaining of diffuse abdominal pain, vomiting and no evacuation either of fecal matter or of flatus. He presented rectal bleeding and constipation since one month. Physical examination revealed abdominal distension and pain to palpation. Mucocutaneous pallor was detected. Abdominal x-ray evidenced air-fluid levels (Figure 1). Laboratory test showed abnormal parameters: anemia with hemoglobine of 8 g/dl, hematocrit of 26% and reticulocyte count of 26 ‰. The patient underwent emergency surgery. Intraoperatively, a tumor of the sigmoid colon had been detected which was infiltrating and stenosing. On exploration, a mass in small bowel diverticulum, 60 cm proximal to the ileocecal valve was encountered. The mass was 5 cm in maximal diameter. A left hemicolectomy and diverticulectomy were performed (Figure 2). The tumor of the colon was mucinous adenocarcinoma pT3N1 (Figure 3). Histopathological diagnosis for the tumoral diverticulum was low grade GIST (Figure 4) and low risk according to Miettinen and Lasoto's scheme. Mitotic count was 3 per 50 high power field. The immunohistochemistry indicated strong staining for ckit/CD117 (Figure 5) and CD34 (Figure 6) while the expression of smooth muscle actin, desmin and S100 protein were negatives.

## Discussion

GISTs are the most common mesenchymal tumors of the gastrointestinal tract [4]. This group of tumors represents about 0,1 to 3% of all gastrointestinal neoplasms. Most of them are located in the stomach and small intestine [5]. They usually develop in a sporadic fashion. However, familial occurrence has also been reported [4].

The diagnosis is based on morphology and immunohistochemistry. CD117 is positive in 95%, CD34 in 40%-50%, smooth muscle actin in 20%-30%, S100 protein and desmin in 10% of cases [6]. Surgery is typically the first step in the treatment of GISTs. Recurrences, metastatic disease or unresectable tumors can be treated with imatinib [7]. GISTs have been reported to occur synchronously with adenocarcinoma, lymphoma and carcinoid [3].

The simultaneous occurrence of GIST and adenocarcinoma is uncommon [4]. In a series of 200 cases of GISTs, studied by Urbanczyk et al, synchronous tumors were present in seven patients including one adenocarcinoma of the colon [8]. Coexisting GISTs are usually detected incidentally during gastrointestinal surgery for carcinoma [9].

The etiology of this association is association is still unknown, but some theories exist: the hypothesis that the association is due to a simple coincidence particulary in areas with high rate of digestive cancer is proposed [10]; a possible explanation is represented by the metallothioneins which protect against DNA damage, apoptosis, cell survival, angiogenesis and oxidative stress [11]. Metallothioneins have been reported to be down regulated in some type of cancers including gastric, colorectal, liver and central nervous system [12]. This theory is supported by nucleolar expression of metallothioneins in GIST [13]; the development of these tumors may involve common carcinogenic agent. Sigimura et al [14] revealed that enteral nitrosoguanidine produces adenocarcinoma in rats. In contrast, simultaneous exposure to both nitrosoguanidine and acetylsalicylic acid causes synchronous development of both gastric cancer and leiomyosarcoma [15].

## Conclusion

The limited number of these cases cannot confirm the existence of a common factor in tumorigenesis of these different tumors. Further studies are needed to clarify the possible association.
